# PAMAM dendrimers as efficient drug nanocarriers of natural compounds in breast cancer therapeutics

**DOI:** 10.3389/fonc.2026.1809604

**Published:** 2026-04-20

**Authors:** Syed Gulfishan, Mir Waqas Alam, Pratibha Pandey, Md Ali Mujtaba, Meenakshi Verma, Sorabh Lakhanpal, Anuj Kumar Rana, Fahad Khan

**Affiliations:** 1 Department of Biomedical Sciences, Division of Pharmacology, College of Medicine, King Faisal University, Al-Ahsa, Saudi Arabia; 2 Department of Physics, College of Science, King Faisal University, Al-Ahsa, Saudi Arabia; 3 University Centre for Research and Development, Chandigarh University, Mohali, Punjab, India; 4 Center for Health Research, Northern Border University, Arar, Saudi Arabia; 5 School of Pharmaceutical Sciences, Lovely Professional University, Phagwara, Punjab, India; 6 School of Applied and Life Sciences, Uttaranchal University, Dehradun, India; 7 Department of Community Medicine, Saveetha Medical College and Hospitals, Saveetha Institute of Medical and Technical Sciences, Chennai, Tamil Nadu, India

**Keywords:** breast cancer, dendrimers, drug delivery, nanocarriers, natural compounds, PAMAM

## Abstract

Breast cancer is among the most prevalent cancers in women and leads to significant mortality worldwide. It remains a global health issue, affecting millions of women annually despite progress in diagnosis and treatment. Advanced-stage breast cancer often lacks effective treatments, and drug resistance commonly contributes to poor patient outcomes. This highlights an urgent need to develop new therapeutic alternatives that are more effective, less toxic, and more affordable. Many medicinal plants are rich sources of novel bioactive compounds, offering valuable sources for exploring antitumor potential. While these natural compounds have shown promise in preclinical studies, challenges related to stability, solubility and bioavailability have limited their clinical use. To overcome these limitations, poly(amidoamine) (PAMAM) dendrimers have emerged as a leading type of nanocarrier. These nanoscale molecules which range in size (~1–100 nm) and configuration, offer a promising strategy for treating various cancers, including breast cancer. This review aims to critically examine the recent studies on the development of PAMAM dendrimers as carriers of natural compounds and evaluated their anticancer potential in preclinical models. Unlike previous reviews, this review uniquely summarizes PAMAM dendrimers for delivery of natural compounds specifically in breast cancer. It also highlighted key areas for future research and potential approaches to advancing PAMAM dendrimers as effective delivery systems for plant-based therapeutics.

## 1 Introduction

Breast cancer, the most common malignancy and leading cause of cancer-related mortality among women globally, affects the lives of millions of women. In 2020, over 2.3 million new breast carcinoma diagnoses occurred, resulting in 685,000 deaths from the disease (1). There is substantial variation in the incidence rates of this disease across various countries and regions globally. In many Asian and African nations, the rates are under 40 per 100,000 women, whereas in Australia/New Zealand, North America, and some regions of Europe, the rates surpass 80 per 100,000 women. Mortality rates exhibited less variability across various geographical regions (2, 3). Nonetheless, nations in transition exhibit a higher rate of breast cancer mortality than those that have completed the transition. By 2040, the yearly incidence of breast carcinoma is estimated to exceed 3 million new cases, leading to 1 million deaths each year as a consequence of population expansion and aging (4). Breast cancer has long been classified as one of the deadliest cancers in terms of incidence and fatality rates (5–7).

Conventional chemotherapeutic strategies remain the primary therapeutic option for several types of malignancies, however multiple challenges such as systemic toxicity, restricted selectivity, and various side effects exist. Cancer therapy mainly utilizes chemotherapy agents or drugs that target fast proliferating cells, resulting in adverse effects on healthy rapidly growing cells, such as hair follicles and the gastrointestinal tract epithelium (8). Many cancer cells increasingly develop resistance to conventional therapies, which is a contributing factor. In case of breast carcinoma, the diagnosis and assessment of the severity of breast cancer determine the necessity for preoperative (neoadjuvant) systemic therapy. Breast cancer management necessitates tailored therapies that are effective and have minimal adverse effects. Significant emphasis should be directed towards diminishing global disparities in access to diagnostics, multidisciplinary treatment, and novel pharmaceuticals, as breast cancer is a worldwide issue (9, 10).

Despite considerable efforts in preclinical environments, minimal advancements have been made in the translation of phytochemicals to human applications (11). A potential factor for clinical translation failure is the poor delivery of promising natural compounds to the target tumor site. Consequently, it is imperative to develop novel and effective delivery methods to overcome these disadvantages (12, 13).

In drug delivery, polymers play a crucial role as nanocarriers for biomacromolecular agents and small drug molecules. Many polymer-based nanomedicines have been developed, and some, including poly(lactic-co-glycolic acid) (PLGA), have received clinical approval for commercialization. Among the numerous polymers utilized for nanocarriers, flexible “dendrimers” have garnered the attention of researchers in the drug delivery domain (14–16).

Dendrimers are innovative, highly branched, three-dimensional polymeric nanocarriers that are created through repeated addition reactions. Dendrimers vary in size from 1.1 nm for 1.0 G to 9.8 nm for 8.0 G. (17, 18). Dendrimers as drug delivery carriers offer advantages over typical polymers, including enhanced water solubility, polyvalency, biocompatibility, and precise molecular weight (19–21). Diverse dendrimers have been engineered and utilized as drug delivery systems for natural products, including polyamidoamine (PAMAM), polylysine (PLL), polypropylene (PPI), and polyglycerol (PG) (22, 23).

Polyamidoamine (PAMAM), the first dendrimer described (24), has garnered significant attention and undergone extensive investigation over the past two decades. Because of their well-regulated synthesis process, they possess the desirable property of low polydispersity (25). Although numerous reviews have addressed PAMAM dendrimers as nanocarriers for cancer therapy, no comprehensive review appears to have specifically examined their use for delivering natural compounds in breast cancer. Most existing reviews address natural compounds (26, 27; or dendrimers-based drug delivery systems independently (28), without systematically examining how PAMAM dendrimers can enhance the delivery and therapeutic efficacy of natural compounds. This review uniquely integrates current knowledge on PAMAM dendrimers-mediated delivery of natural compounds in breast cancer, highlighting their potential to address the challenges of poor pharmacokinetics associated with natural compounds.

## 2 Methodology

A comprehensive literature search was performed to identify the relevant studies investigating the use of PAMAM dendrimers as nanocarriers for natural compounds in breast cancer. Main electronic databases including PubMed, Web of Science, Scopus, and Google Scholar were systematically searched for articles published up to 2025 using combinations of relevant keywords such as PAMAM dendrimers, poly(amidoamine) (PAMAM) dendrimers, natural compounds, phytochemicals, plant derived compounds, drug delivery, nanocarriers, cancer therapy, breast cancer. Studies were included if they reported the use of PAMAM dendrimers as delivery systems for natural compounds with anticancer activity and provided preclinical evidence in *in vitro*/*in vivo* breast cancer models. Articles unrelated to PAMAM dendrimers, studies involving synthetic drugs, insufficient experimental analysis, conference abstracts, book chapters, and editorial were excluded.

## 3 Natural compounds-based breast cancer therapy and their challenges

Plant-derived natural compounds have emerged as prospective antitumor agents due to their capacity to specifically target various hallmarks of breast cancer while demonstrating reduced systemic toxicity compared to traditional therapy (29). Phytochemicals including polyphenols, alkaloids, terpenoids, and sulfur-based compounds, have the ability to exert antiproliferative, apoptotic, immunomodulatory, anti-metastatic and antiangiogenic effects (30). These anticancer effects are typically achieved by modulating crucial pathways, such as NF-κB, PI3K/AKT/mTOR, and Wnt/β-catenin (31–33). When compared to traditional breast cancer treatments, phytochemicals offer several advantages, including the ability to target numerous oncogenic pathways concurrently, overcome drug resistance, show less toxicity, and offer multitarget approach. For example, curcumin, genistein, resveratrol, and sulforaphane have the ability to simultaneously target numerous signaling pathways involved in breast cancer progression, which provides a more comprehensive and efficient approach to the prevention and treatment of cancer (34).

These phytochemicals influence many pathways associated with apoptosis, metastasis, and the immunological response (35, 36). Additionally, phytochemicals have the potential to be utilized as adjuvants, which can reduce the adverse drug reactions and improve the overall quality of life of patients (37). Furthermore, phytochemicals have the ability to influence the tumor microenvironment, a multifaceted and ever-changing setting that modulates tumor cell proliferation, aberrant tumor vasculature development, and altered energy metabolism (38). Phytochemicals influence the tumor microenvironment through the remodeling of the extracellular matrix or by modulating the synthesis of cytokines, chemokines, and growth factors, thereby resulted in inhibition of cancer cell proliferation and metastasis (39). Their antitumorigenic effects are achieved through the suppression of immunosuppressor cells or the activation of cytotoxic T lymphocytes. Despite the extensive testing of numerous substances in both *in vitro* and *in vivo* research, only a small percentage advances to clinical application (40).

**Figure 1:**
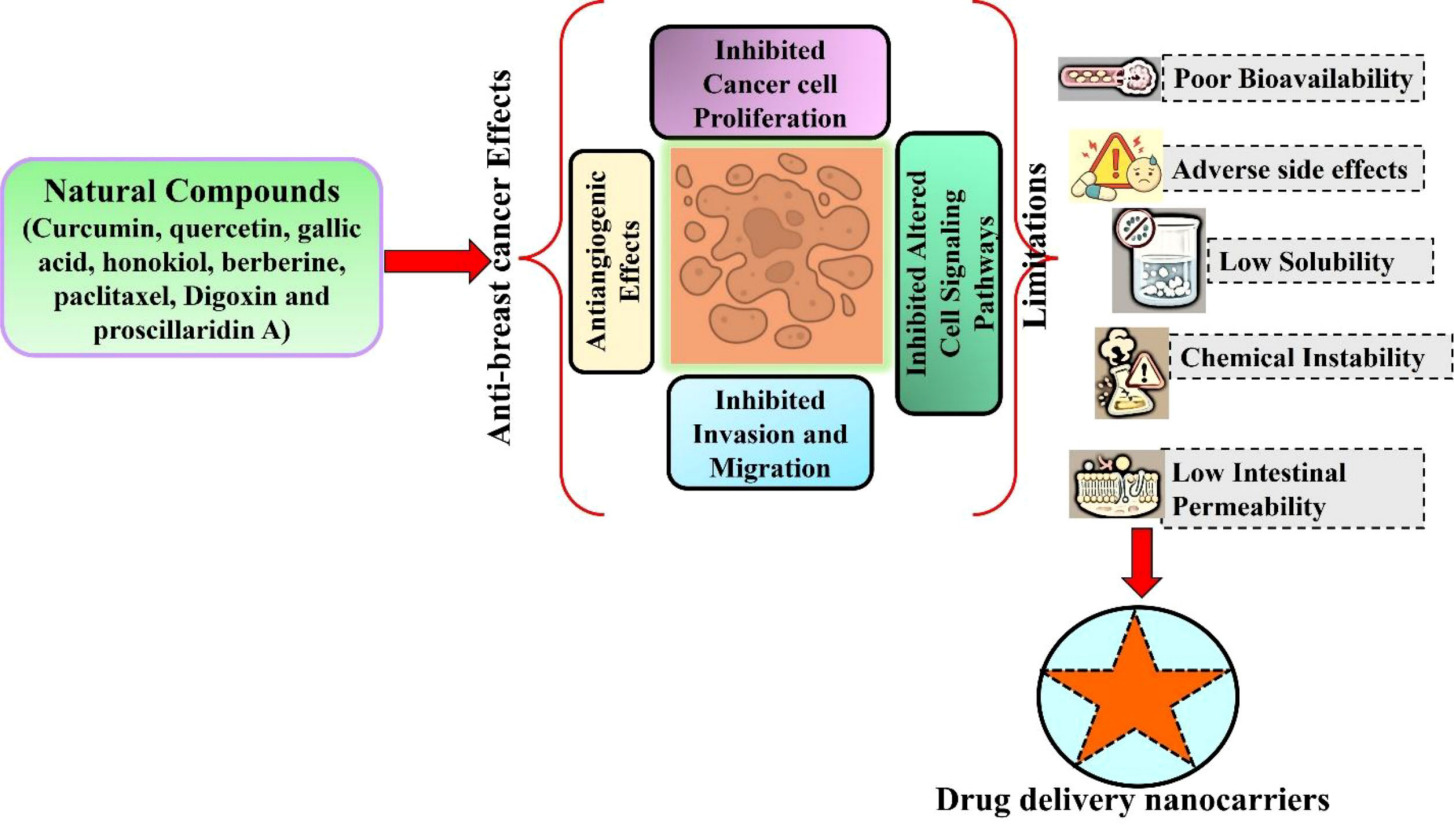
Anticancer effects of natural compounds in breast cancer cells and their key limitations associated with therapeutic applications. Natural compounds such as curcumin, quercetin, gallic acid, berberine, honokiol, digoxin exhibit promising anticancer activities by inhibiting cell proliferation, repressing angiogenesis, inducing apoptosis and modulating cell signaling pathways. However, their clinical use is limited by poor bioavailability, adverse effects, solubility issues, and inadequate absorption, highlighting the need of advanced drug delivery nanocarriers to enhance therapeutic effects in breast cancer.

Phytochemicals have strong preclinical anticancer activity and a variety of pharmacological actions, but they encounter significant obstacles that prevent them from being clinically translated for breast cancer therapy. A significant obstacle hindering the practical translation of natural compounds is their intrinsically low systemic bioavailability, primarily due to adverse physiochemical characteristics (41, 42) (**Figure 1**). This is mostly due to low aqueous solubility, frequently associated with high lipophilicity, as indicated by increased LogP values. For example, effective anticancer compounds such as curcumin (LogP ~3.3–3.6), resveratrol (LogP ~3.1), and quercetin (LogP ~2.0) demonstrate significant *in vitro* efficacy but do not attain therapeutic plasma levels due to restricted dissolution and absorption (43, 44). It should be noted that although solubility is a key barrier, many natural compounds have low systemic exposure due to a combination of factors like substantial first-pass metabolism and decreased intestinal permeability (45, 46).

They are also severely limited by their ineffective tumor accumulation and non-specific biodistribution. This poses significant challenges for solid malignancies such as breast cancer, which exhibit aberrant vasculature and a complicated tumor microenvironment (47). Most phytochemicals are ineffective because to their non-specific distribution and absence of active targeting mechanisms, which prevent them from achieving therapeutic concentrations in tumor tissues and lead to increased systemic dispersion (48, 49). Taken together, these constraints make it harder to move phytochemicals from the lab to the clinic.

Curcumin and resveratrol, although exhibiting significant *in vitro* effects, show minimal plasma concentrations following oral administration due to swift hepatic metabolism and elimination, thereby severely limiting their *in vivo* therapeutic efficacy (50, 51). Furthermore, numerous phytochemicals have swift metabolic breakdown, leading to a brief plasma half-life and restricted presence at the tumor site (52).

Moreover, dietary changes can reduce the risk for most cancers (53). The predominant herbal active compounds are hydrophobic and exhibit low solubility. The limited therapeutic application of herbal remedies arises from the inadequate solubility and hydrophobic nature of their active constituents, leading to diminished bioavailability and heightened systemic clearance, hence requiring frequent administration or elevated dosages. Nonetheless, nano or micro formulations can mitigate these challenges. Diverse polymeric or lipid carriers are present in nanocarriers or sustained-release formulations, employed for drug delivery via several routes, including transdermal, buccal, oral, and parenteral administration. They enhance therapeutic efficacy and localization at the intended target, hence improving patient compliance (54). One example is the use of oral polymeric nanoparticles to improve *Cuscuta chinensis*’s low water solubility (55). The inadequate water solubility and adverse effects of camptothecin can be alleviated through various nanoformulations such as polymer conjugate hydrogels, liposomes, polymeric nanoparticles, or solid lipid nanoparticles (56, 57).

## 4 Dendrimers as drug delivery agent

The pharmaceutical industry has been significantly impacted by advancements in nanotechnology, particularly through the development of nanosystems for drug and gene delivery. These nanosystems often take advantage of the enhanced permeability and retention (EPR) effect, which allows drugs to accumulate more readily in tumor tissues due to its leaky blood vessels and poor lymphatic drainage (58). However, many conventional nanocarriers are limited by low drug-loading capacity and complex production methods. Dendrimers have emerged as a versatile alternative in recent years (59, 60). The name originates from the Greek word ‘dendron’ meaning tree, reflecting their branching architecture. Structurally, dendrimers are built from a central core to which successive layers of branches are added, ultimately forming a symmetrical, spherical molecule (61–63). They mainly consist of three main parts: a core, branching layers, and terminal functional groups (64, 65). Compared to traditional linear polymers of similar molecular weight, dendrimers can display a wider range of functional properties (66–68).

Dendrimers can be categorized according to their chemical composition, function, generation number, and other criteria. Common types include polyamidoamine (PAMAM), poly(propylene amine) (POPAM), and poly-L-lysine (PLL) dendrimers (69, 70). Hybrid dendrimers also exist, consisting of a core from one type of dendrimer and branches from another (71). Researchers can further modify dendrimers to acquire specific traits, such as pH sensitivity. For example, pH-sensitive dendrimers are designed to release their therapeutic cargo in acidic environments like those found in tumors. In studies using cells and animal models, these modified dendrimers have effectively targeted tumors with chemotherapy drugs, substantially reducing damage to healthy cells (72).

Dendrimers can encapsulate, covalently link, or interact electrostatically with anticancer medications, which can be stored either within the dendrimers or on functional groups located on their surface (73, 74). The targeted distribution of dendrimers is essential in cancer cases to minimize adverse reactions on healthy tissues, including the bone marrow and internal organs, which can occur when chemotherapy drugs are administered freely (75). For more precise targeting, dendrimers can be coupled with pharmaceuticals and targeting molecules, such as folic acid, monoclonal antibodies, or other peptides (76, 77). Furthermore, poly9ether hydroxylamine) dendrimers have been used to enhance the aqueous solubility of poorly soluble anticancer drugs (78, 79).

Dendrimers can be delivered to specific targets via active or passive mechanisms (80–82). The passive mechanism relies on the accumulation of PEGylated dendrimers in cancerous tissues, which is attributed to the permeability and retention effects (83). Tumors exhibit aberrant vascularization due to tumor angiogenesis, and inadequate lymphatic drainage results in the deposition of dendrimers macromolecules in the tumor microenvironment (84, 85). Active targeting mechanism is accomplished by conjugating drug-loaded dendrimers with various specific targeting molecules that facilitate interactions with specific cell receptors (86, 87). These distinct characteristics of dendrimers including nanoscale size, high branching network makes them as ideal drug delivery candidate.

## 5 PAMAM dendrimers

**Figure 2:**
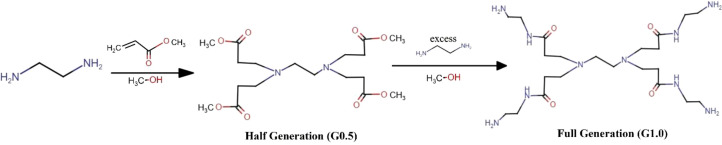
Synthesis of PAMAM: PAMAM is synthesized from amino and amido groups, created by reacting the core with α-β unsaturated acrylates through Michale addition and amidation reactions. Michael addition alkylates the core to form a half-generation ester terminated group (G0.5). The next step involves amidating the ester group using an excess of the core molecules and subsequently generated an amine-terminated first/full generation PAMAM (G1.0) These two procedure can be repeated iteratively to create higher generations of PAMAM.

Polyamidoamine (PAMAM) dendrimer was initially synthesized and described by Tomalia et al. in 1985 (24), with its dendritic architecture established through the incorporation of repeating branching units and numerous reactive groups affixed to the outer surface (88). PAMAM dendrimers are spherical polymers characterized by monodispersity and are one of the most extensively investigated polymeric molecules (89). Its architecture comprises a central core from which repeating units extend outward and terminal groups attached to the peripheral repeating units, resulting in a three-dimensional configuration identical to that of certain spherical proteins (90) (**Figure 2**). The number of generations (G) that make up these dendrimer molecules is the first generation, which includes the first through seventh generations (G1-G7) and is the first full series of dendrimeric molecules (91).

PAMAM dendrimers have been evaluated as carriers for various molecules and have demonstrated significant potential in diagnosis and therapy because of their ability to enhance solubility, bioavailability, absorption and targeted distribution of drugs. For example, Gallien et al., investigated the use of PAMAM dendrimers as nanocarriers for curcumin. Their study showed that encapsulation of curcumin in the dendrimer significantly reduced the viability of glioblastoma cells, whereas the effect on non-cancerous cells was minimal. This suggests that dendrimers-based delivery may enhance the selective anticancer activity of curcumin while limiting toxicity to normal tissues (92). Another study by Kianamiri et al. demonstrated improved intracellual transport and enhanced anticancer activity of triphenylphosphonium conjugated curcumin PAMAM formulation in hepatocellular carcinoma cells (93).

In addition, their toxicity can be assessed concurrently across various cell lines (94). Researchers have observed that the cytotoxicity of PAMAM dendrimers is primarily based on the number and type of functional groups on the outer layer. For instance, cationic PAMAM dendrimers generally demonstrate significant toxicity, whereas anionic and neutral types display moderate to non-toxic effects (95). PAMAM dendrimers interact with the tumor microenvironment (TME), which is characterized by acidic pH, hypoxia, stromal barriers, and a redox environment, facilitating the delivery of anticancer agents (96). Their nanoscale dimensions facilitate accumulation in tumor tissues via the increased permeability and retention (EPR) effect. Recent advancements have integrated pH-responsive release mechanisms that utilize the acidic TME to initiate localized drug release, hence improving the therapeutic index and reducing systemic exposure (97). PAMAM dendrimers can be altered with hypoxia-sensitive linkers such as azobenzene, which initiate drug release in hypoxic TME (98). Moreover, their reduced dimensions and surface modifications by acid-sensitive or ultrasound-sensitive linkers facilitate increased distribution across stromal barriers and the extracellular matrix which leads to improved targeted drug delivery and therapeutic effects in cancer treatment (99, 100).

PAMAM dendrimers can efficiently encapsulate anticancer agents and release them in response to specific physiological or chemical stimuli. This design facilitates drug release at tumor cell locations, thus mitigating the effects on healthy cells. Bai et al. synthesized pH-responsive docetaxel loaded PAMAM nanoparticles for the treatment of lung cancer-induced bone metastasis. The results of *in vitro* findings demonstrated that these nanoparticles markedly improved the anticancer efficacy of docetaxel and effectively suppressed osteoclastogenesis. Docetaxel loaded PAMAM nanoparticles accumulates at the site of bone metastasis in mice model to suppress bone resorption, alleviate pain response, and suppress the progression of metastasis (101). When synthesizing PAMAM dendrimers, materials exhibiting lower toxicity should be considered, or toxicity can be minimized through appropriate modifications. For instance, decreasing the level of PEGlyation or employing specific dendritic architectures can mitigate the negative impact on healthy cells (102). Furthermore, many PAMAM dendrimers exhibit biological activity, antimicrobial activity, or selective toxicity against cancer cells, while having no effect on healthy cells (103).

In subsequent section, we have summarizes the recent advancements of PAMAM as nanocarriers for natural compounds in breast cancer. This study aims to provide innovative viable strategies for the advancements of PAMAM dendrimers based nanocarriers for breast cancer treatment through the utilization of natural compounds.

## 6 PAMAM dendrimers as natural compounds nanocarriers in breast cancer

**Figure 3:**
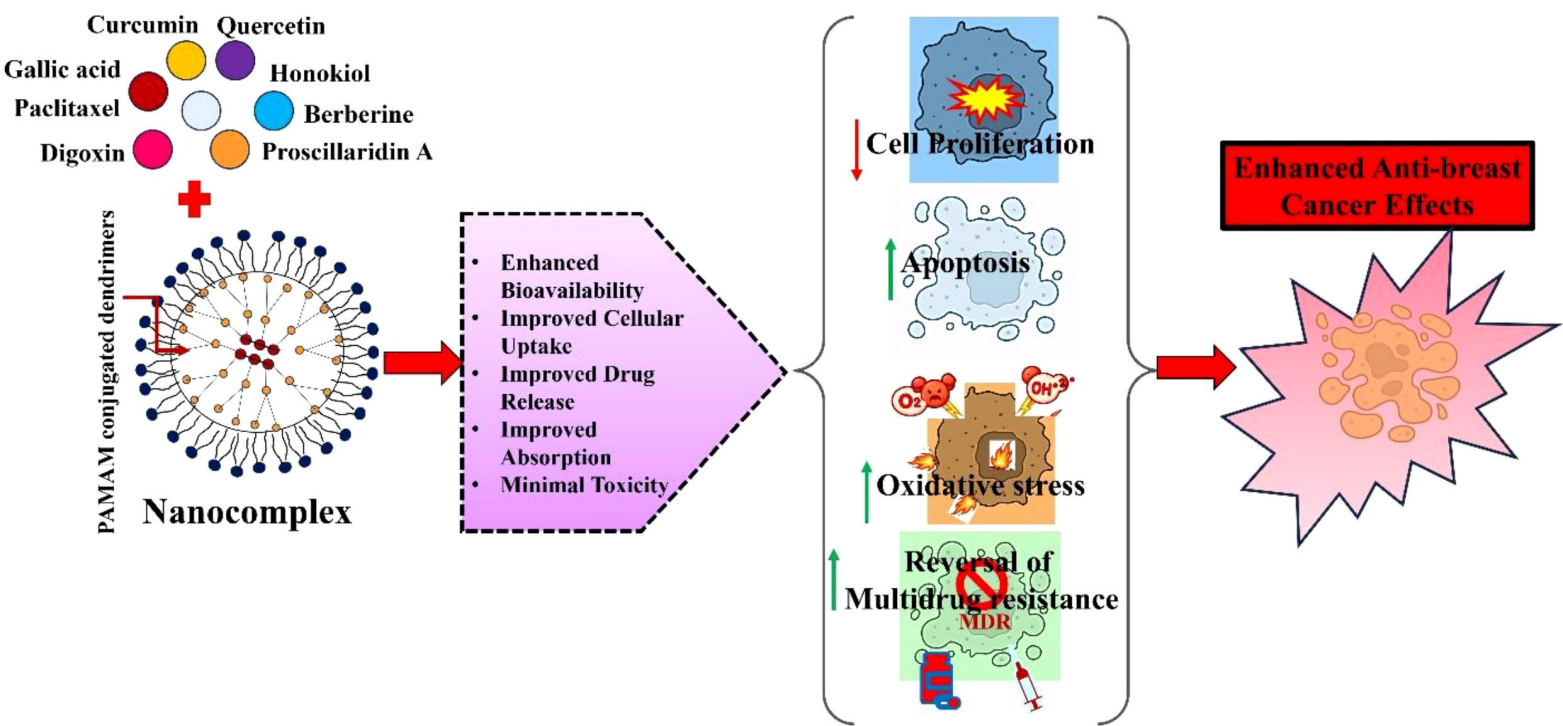
Natural compounds based PAMAM dendrimers for enhanced anticancer efficacy in breast cancer models. PAMAM dendrimers enables efficient encapsulation of conjugation of natural compounds such as gallic acid, curcumin, gallic acid, honokiol, berberine. These nanocarriers enhanced oxidative stress mediated apoptosis and antiproliferative effects of natural compounds in *in vitro* and *in vivo* models of breast cancer.

There is mounting evidence that plant-derived compounds have therapeutic potential in the treatment of various carcinomas. However, majority of natural compounds exhibit low solubility and limited bioavailability, presenting considerable obstacles to their development as clinical drugs. To address these concerns, several nanocarriers have been developed (104, 105). PAMAM dendrimers are the best choice for transporting natural compounds because of their many advantages (**Figure 3**) (106). PAMAM dendrimers have shown significant promise in cancer therapy using natural compounds, primarily because of their capacity to improve the bioavailability, pharmacokinetics, and pharmacodynamics of active therapeutic agents (**Table 1**). This highlights the need for additional research on the optimization of internalization mechanisms and the cytotoxic effects of these nanocarriers (124).

**Table 1:** Preclinical evidences of natural compounds and PAMAM dendrimers based nanocarriers showing improved antitumor effects in breast cancer.

Natural compounds	Class	Nanocarriers	Cancer model (*In vitro*/*in vivo*)	IC_50_	Anticancer target/mechanism	Reference
Curcumin	Polyphenol	PAMAM dendrimers	T47D cells	10.5 μM	Enhanced anticancer effects by inhibiting telomerase activity, improved solubility and cellular uptake	(107)
Curcumin	Polyphenol	PAMAMG0.5 dendrimers	MCF-7 cells	–	Enhanced cytotoxic effects, superior encapsulation and loading capacity	(108)
Curcumin	Polyphenol	PAMAMG5 dendrimers	MCF-7 cells	–	Enhanced anticancer effects; improved loading capacity, encapsulation efficiency and pH sensitive drug release	(109)
Curcumin	Polyphenol	PAMAM-G2	MCF-7 cells	–	Enhanced anticancer effects and drug loading capacity	(110)
Curcumin	Polyphenol	Methoxy-PEGylated PAMAM generation 3 dendrimers	MCF-7 cells	–	Enhanced anticancer and apoptosis inducing effects; stable drug loading capacity, encapsulation efficiency and pH sensitive drug release	(111)
Quercetin	Polyphenol	PEGylated PAMAMG4dendrimer-Margetuximab	MDA-MB-231 cells	100 nM	Enhanced anticancer and apoptosis inducing effects; stable drug loading capacity and controlled drug release	(112)
Quercetin	Polyphenol	PAMAM G4 dendrimer	MCF-7 cells	100–200 nM	Enhanced apoptosis inducing effects via increased ROS generation, caspase activation, Bax and p53 expression; stable drug loading capacity and pH responsive drug release	(113)
Quercetin	Polyphenol	GO-PAMAM	MDA-MB-231 cells	39.41 µg/mL	Enhanced cytotoxic effects; stable drug loading capacity and pH responsive drug release	(114)
Quercetin	Polyphenol	G3, G4 s-triazine-based dendrimers	MCF-7 cells	12.69-29.316 µM	Enhanced anticancer and apoptosis inducing effects via downregulation of VEGF levels; stable drug loading capacity, efficient drug encapsulation and controlled delivery	(115)
Gallic acid	Polyphenol	PAMAM G4 dendrimer	MCF-7 cells	25 μg/mL	Enhanced cytotoxic effects	(116)
Honokiol	Polyphenol	PAMAM G4 dendrimer	4T1 cells and 4T1 xenograft mice model	2.2 μg/mL	Improved cytotoxicity and cellular uptake	(117)
Berberine	Alkaloid	PEGylated PAMAM dendrimer	MCF-7 cells	–	Enhanced cytotoxic effects; improved loading capacity, encapsulation efficiency and controlled drug release	(118)
Berberine	Alkaloid	PAMAM G4 dendrimer	MCF-7 and MDA-MB-468 cells	2.79-4.97 μg/mL	Enhanced anticancer effects; Efficient encapsulation, conjugation and sustained drug release	(119)
Paclitaxel	Alkaloid	cationic PAMAM dendrimers	MDA-MB-468 cells and mouse breast cancer cell line 4T1; 4T1 metastatic mouse model	–	Inhibited primary tumor growth and metastasis; Efficient encapsulation, and sustained drug release	(120)
Paclitaxel	Alkaloid	Phospholipid-modified PAMAM dendrimer	MCF-7/ADR cells	–	Increased apoptosis, reversible of drug resistance, and cell cycle arrest; Efficient conjugation, and sustained drug release	(121)
Paclitaxel	Alkaloid	PAMAM dendrimer	MCF-7 cells	80 nM	Increased cytotoxic and apoptotic effects; efficient cellular uptake	(122)
Digoxin and	Glycosides	G3 PAMAM-NH(2)	MCF-7 and MDA-MB-231 cells	88 ± 2 nM and 105 ± 2 nM	Improved apoptotic activity; improved loading capacity, encapsulation efficiency and controlled drug release	(123)
Proscillaridin A	Glycosides	G3 PAMAM-NH(2)	MCF-7 and MDA-MB-231 cells	24 ± 3 nM and 43 ± 2 nM	Improved apoptotic activity; improved loading capacity, encapsulation efficiency and controlled drug release	(123)

PAMAM dendrimers enhance the anticancer activity of medicinal drugs through multiple cellular and cellular pathways. A key examples of these pathways is enhance cellular uptake. By interacting with the negatively charged phospholipids on cancer cell membranes, the positively charged terminal amine groups on PAMAM dendrimers enhance uptake through endocytosis pathways such as micropinocytosis and clathrin-mediated endocytosis. This process enhances cytotoxicity in tumor cells and increases drug concentration (125, 126). Another important mechanism consists of improving the solubility and stability of inadequately water-soluble anticancer compounds, particularly natural compounds such as curcumin and resveratrol. The interior cavities and functional groups of PAMAM dendrimers facilitate drug encapsulation via hydrogen bonding, and hydrophobic and electrostatic interactions, subsequently improving water solubility and protecting against degradation (127).

PAMAM dendrimers promote passive tumor targeting via the increased permeability and retention (EPR) effect. The accumulation of dendrimer-drug complexes within tumors is favored by the leaky vasculature and inadequate lymphatic drainage of tumor tissues. Subsequently, this resulted in increased drug concentrations at the tumor site and reduced toxicity to healthy cells (128).

Moreover, surface functionalization with targeting ligands, such as folic acid or triphenylphosphonium, facilitates selective delivery to cancer cells or specific organelles. This process also potentially improves the efficiency of PAMAM dendrimers encapsulated anticancer drugs (129).

### 6.1 PAMAM dendrimers for curcumin delivery

Curcumin a polyphenolic compound, is derived from the rhizome of *Curcuma longa* Linn. Plant. The antitumor properties of curcumin and its analogues have been comprehensively documented and are of considerable importance in the treatment of various malignancies including breast cancer (130, 131). Besides its significant anticancer potential in preclinical models, curcumin has some disadvantages, including low water solubility and inadequate ingestion. To address these issues, scientists have attempted to improve curcumin solubility, absorption, and bioavailability by using nanotechnology-based drug delivery systems (132). In this regard, PAMAM dendrimers have garnered considerable attention for their capacity to enhance the bioavailability of curcumin, thereby improving the efficiency of cancer treatment (133). To enhance the effectiveness of curcumin in breast cancer cells, PAMAM dendrimers were developed to encapsulate curcumin nanocarriers, their cytotoxic effects were evaluated in T47D cells. The findings of this study clearly show that these dendrimers containing curcumin significantly inhibited the proliferation of T47D cells (107).

Falconieri et al., developed G5 PAMAM dendrimers with curcumin to boost its bioavailability of and assessed their cytotoxicity in human breast cancer MNF-7 cells. The study revealed that the G5 PAMAM dendrimers of curcumin exhibited improved solubility, drug release and cytotoxicity in MCF-7 breast cancer cells (108). Nosrati et al., synthesized curcumin loaded G5 PAMAM dendrimers encapsulated with citric acid coated Fe_3_O_4_ nanoparticles and assessed their cytotoxicity and antitumor efficacy against breast cancer cells. They observed that these nanocarriers offer a more effective strategy for controlled and gradual release of curcumin in human breast cancer (MCF-7) cells. Furthermore, these dendrimers demonstrated stronger anticancer potential against MCF-7 cells than curcumin alone (109).

Ramzshoar et al. developed a PCL/PAMAM/curcumin nanofibers and evaluated their antitumor effects against breast cancer MCF-7 cells. The study revealed that an increase in dendrimer concentration (15-35%) resulted in enhanced curcumin loading efficiency and improved inhibition rate (51.5%-42.4%) of cancer cell. These findings underscore the efficacy of this dendrimer based nanoscaffold for effective curcumin delivery (110).

In order to facilitate thermochemotherapy, Montaberabadi et al. created a nanocomplex using methoxy-PEGylated PAMAM G3 dendrimers and SPIONs. The nanocomplex demonstrated enhanced cytotoxicity and apoptotic cell death in breast cancer (MCF-7 cells) over expressing folate receptors than in cells with low receptor levels. Moreover, this nanocomplex demonstrated the capacity to extend the release profile of curcumin, thereby significantly overcoming its limited bioavailability (111, 134). These reports clearly indicated that different generations of PAMAM dendrimers encapsulated curcumin increased antitumor potential of curcumin against different types of breast cancer cells.

### 6.2 PAMAM dendrimers for quercetin delivery

Quercetin, a prominent flavonoid, is extensively documented for its wide range health advantages, including anti-inflammatory, antidiabetic, antiviral, and antitumor properties (135–137). Among the many flavonoids, quercetin has drawn attention due to its possible role in preventing breast cancer pathogenesis. This can be achieved through various mechanisms, such as enhancing immune response, reducing oxidative stress, apoptotic induction, suppressing metastasis, invasion, and modulating oncogenic cell signaling pathways (138, 139). Numerous reports have investigated to enhance antitumor efficacy of quercetin by employing drug delivery systems such as nanocarriers and drug-loaded polymers. This, led to the effective synthesis of several PAMAM dendrimers for delivering quercetin to preclinical breast cancer models (140).

In this context, Khakinahad et al. developed PAMAM G4 dendrimers coupled with margetuximab and polyethylene glycol to deliver quercetin to breast cancer MDA-MB-231 cells. The findings revealed that quercetin nanoscaffold can effectively load drug and regulate drug release in *in vitro* environment. The MTT assay indicated that cell viability decreased to 14.67% at a dose of 800 nM following 24 h of exposure with this nanocomplex. Moreover, this dendrimer nanocomplex caused cell cycle arrest and a substantial upregulation in expression levels of apoptotic markers in breast cancer cells (112).

A recent study by Baghersad and Salimi, created an innovative polycaprolactone-polyamidoamine linked PAMAM G4 dendrimer nanocarrier that effectively co-delivered silver nanoparticles and quercetin to MCF-7 breast cancer cells. The silver nanoparticles and quercetin-encapsulated PAMAM dendrimers demonstrated 14% cell viability in MCF-7 cells after 72 h exposure at a dose of 400 nM. qRT-PCR analysis confirmed the apoptosis-inducing potential of the PAMAM nanocomplex through the elevated expression levels of caspase, P53, and Bax genes. Additional findings revealed that the nanocomplex stimulated ROS production and cell death MCF-7 breast cancer cells (113).

Matiyani et al., evaluated the efficacy of PAMAM dendrimer functionalized graphene oxide (GO) nanocarriers for site specific delivery of quercetin in MDA-MB-231 breast cancer cells. The investigation revealed that these nanocomplex dendrimer exhibited a superior quercetin loading capability compared to that of GO. Additionally, synthesized dendrimer nanoscaffold demonstrated both regulated and pH-sensitive release of quercetin. Moreover, GO-PAMAM exhibited non-toxicity towards HEK 293T cells, whereas quercetin loaded PAMAM dendrimer a exhibited a significant cytotoxic effect on MDA-MB-231 cells (114).

Furthermore, Ramadan et al., synthesized novel, logically engineered G3/G4 dendrimers for quercetin delivery, which exhibited MMP-2/9 inhibitory activity to effectively arrest the progression of both breast and liver cancers while minimizing off-target adverse reactions. Quercetin nanocomplex also promoted apoptosis by downregulating the expression of VEGF and MMP-9 in both cell types. These results further highlight the potential of PAMAM nanoscaffolds as quercetin drug delivery vehicles due to its pharmacophoric properties that can be tailored to target and destroy tumor tissues (115).

### 6.3 PAMAM dendrimers for gallic acid delivery

Gallic acid is a polyphenol molecule with many biological effects including antioxidant, anti-inflammatory, anticancer, and antimicrobial properties (141, 142). Recent studies have substantiated the antitumor properties of gallic acid, indicating its potential as an anticancer drug molecule (143–145). Simultaneously, an increasing number of research indicated that gallic acid had substantial anticancer efficacy against preclinical breast cancer models (146–148).

However limited bioavailability and rapid systemic clearance of gallic acid obstructed its therapeutic applications. To address this issue, nanotechnology based novel delivery systems may be beneficial for cancer therapy. In this context, PAMAM dendrimers serve as advanced and effective vehicles for drug delivery. To develop a cancer-targeted drug delivery system, Sharma et al., created a 4.0 G PAMAM dendrimer coupled with gallic acid and studied its cytotoxic effects in MCF-7 breast cancer cells. The results showed that the gallic acid coupled PAMAM nanocomplex had an IC_50_ value of 25 μg/mL against the MCF-7 cell line and over 100 μg/mL against the normal cell line. The cytotoxicity investigation showed that the nanocomplex had four times the selectivity over normal cell lines and was more active against the breast cancer cell line (MCF-7). The 4.0 G PAMAM and gallic acid nanoconjugate may exhibit synergistic activity when combined with chemotherapeutic drugs (116).

### 6.4 PAMAM dendrimers for honokiol delivery

Honokiol is a low molecular weight biphenyl lignan with multiple health benefits, derived from the bark of Magnolia officinalis or related species within the Magnoliaceae (149). Clinical investigations have indicated that honokiol administration induces anti-inflammatory, antioxidative, antimicrobial, neurological, antiangiogenic and antitumor activity (150–152). Furthermore, safety and toxicological assessments of honokiol have not revealed any substantial toxicity (153, 154). In recent years, the anticancer properties of honokiol have attracted considerable attention from researchers. Studies reported that honokiol inhibited cancer cell invasion and metastasis by modulating several signaling pathways such as, PI3K/Akt/mTOR, EGFR, and NF-κB (155–157). It also demonstrated that honokiol effectively induced apoptosis by promoting mitochondrial dysregulation and endoplasmic reticulum stress (158, 159). Other research has indicated that honokiol may suppress the migration of breast cancer cells by modulating nitric oxide and cyclooxygenase-2 levels (160). On the other hand, the presence of phenolic hydroxyl groups in its molecular structure, honokiol has limited aqueous solubility and reduced bioavailability, hindering its clinical application in cancer therapy (161). It is vital to develop new techniques to increase the bioavailability and solubility of honokiol (162).

A notable example of nanocarriers utilizing dendrimers is PAMAM dendrimers, which have emerged as a novel drug delivery system due to their exceptional drug loading and release capabilities. A research study conducted by Guo et al., employed oligoethylene glycol linked G4 PAMAM nanocarriers to prepare honokiol loaded nanoparticles, and subsequently optimized them for drug loading efficiency and release kinetics. Furthermore, the cytotoxic potential of these nanoparticles was assessed in *in vitro* and *in vivo* 4T1 breast cancer models. The honokiol nanocomplex exhibited increased cytotoxicity towards 4T1 breast cancer cells, and the cellular uptake mechanism was identified as caveolae- and clathrin-mediated endocytosis. Furthermore, it is worth noting that these nanocomplex of honokiol showed strong anticancer activity in *in vivo*, with no major adverse effects being reported. Additionally, *in vivo* investigations demonstrated that honokiol nanoparticles exhibited superior tumor accumulation compared to HCPT injection (117).

### 6.5 PAMAM dendrimers for berberine delivery

Berberine is an alkaloid isolated from the rhizome, root and stem bark of medicinal plants such as Berberis vulgaris (163, 164). Numerous recent investigations have demonstrated that berberine exhibits diverse pharmacological properties, including anti-inflammatory, antioxidant, antihypertensive, antidiabetic, immunomodulatory, cardioprotective and neuroprotective properties (165–169). In addition, berberine may inhibit and treat various types of tumors caused by genetic modifications and environmental risk factors (170, 171). Accumulating evidence indicates that berberine suppresses proliferation, cell cycle progression, and promotes apoptosis in many cancer cell lines while exhibiting minimal cytotoxicity towards normal epithelial cell lines (172–174). Due to its notable efficacy in preventing, inhibiting, and reversing the progression of various cancer types, berberine has garnered considerable attention as a promising antitumor agent for the treatment of numerous cancers, particularly breast cancer (175).

Besides these advancements, clinical implementation is impeded by insufficient tumor accumulation, rapid systemic clearance, and reduced bioactive concentrations resulting from significant metabolic breakdown. One promising strategy to overcome these limitations and enhance the anticancer effectiveness of berberine is the selective application of the nanocarriers and other drugs in conjunction with berberine (176). Yadav et al. recently synthesized a PEGlyated berberine dendrimer using a biotinylation cross-linking approach. The synthesized berberine dendrimer nanocarriers were characterized with regard to their nanoscale dimensions, and their cytotoxic effects were assessed against breast cancer MCF-7 cells. The berberine dendrimer nanocomplex demonstrated superior cytotoxic efficacy in breast cancer (MCF-7) cells compared to pure berberine (118).

In a separate study, Gupta et al. developed berberine-conjugated G4 PAMAM dendrimers for targeted delivery in breast carcinoma cells. MTT findings demonstrated substantially enhanced anticancer effects of berberine nanoformualtion towards MCF-7 and MDA-MB-468 breast cancer cell lines. Even after 24 h, the time dependent ex vivo hemolytic toxicity of this formulation was less than 5%, suggesting that the formulations were harmless and biocompatible (119).

### 6.6 PAMAM dendrimers for paclitaxel delivery

Paclitaxel is a taxane extracted from the bark of Taxus brevifolia that was firstly characterized in 1971 (177). The first-generation drug formulation Taxol^®^ is widely recognized for its efficacy in enhancing overall survival rates and progression-free survival among cancer patients (178, 179). It received approval for the treatment of multiple malignancies, including breast cancer. Even though paclitaxel has therapeutic advantages, its usage in clinical settings may be restricted due to unfavorable side effects or insufficient pharmacodynamic characteristics. Numerous nanotechnology-based formulations have been proposed for the advancement of paclitaxel delivery that not only enhance the bioavailability of drug but also reduce the adverse reactions related to taxol (180, 181).

For example, PAMAM-based nanocomplexes have been developed to facilitate the effective delivery of paclitaxel in preclinical breast cancer models. Cationic PAMAM dendrimers coupled with drug-binding dodecyl and diethylethanolamine groups demonstrated optimal size, drug loading capacity, cytotoxicity, cfNA binding affinity, and anti-inflammatory efficacy during *in vitro* structural optimization. Paclitaxel-encapsulated nanoparticles diminished serum concentrations of cell-free nucleic acids and inflammatory mediators relative to paclitaxel monotherapy, while concurrently suppressing the progression of primary tumors and their metastasis in *in vivo* tumor model. Furthermore, no notable adverse effects were observed in the serum or primary organs (120).

Liu et al. developed a phospholipid-linked PAMAM nanoparticle delivery system encapsulating a functional siRNA directed against the MDR1 gene to mitigate multidrug resistance in human breast cancer MCF-7/ADR cells. This nanoformulation demonstrated improved cellular absorption of siMDR1, reduced p-gp expression, increased cellular retention of doxorubicin, and hindered cancer cell migration compares to siMDR1 and dendriplexes (PAMAM-siMDR1). Furthermore, the paclitaxel dendrimer nanocomplex demonstrated synergistic activity with paclitaxel in the treatment of multidrug resistance, resulting in enhanced cellular apoptosis and cell cycle regulation (121).

PAMAM dendrimers were also used to carry paclitaxel to carry paclitaxel and miR-21 inhibitors for use in combination treatment against MCF-7 breast cancer cells. The IC_50_ values for the paclitaxel drug dendrimer nanocomplex were markedly reduced to a larger extent in the miR-21 inhibitor transfected cells compared to those administered with paclitaxel alone. Paclitaxel exposure also elevated the proportion of apoptotic cells in miR-21 inhibitor-transfected cells compared to that in control cells. Moreover, the administration of the anticancer agent paclitaxel to miR-21 inhibitor-transfected cells led to a marked decrease in cell survival and invasion relative to that in control cells (122).

### 6.7 PAMAM dendrimers for digoxin and proscillaridin A delivery

Cardiac glycosides are a category of organic chemicals characterized by a glycoside component and an aglycone (steroid) component. These compounds are used to manage cardiac disorders, including ischemia, congestive heart failure, and cardiac arrhythmia (182). Numerous studies have indicated the potential anticancer properties of cardiac glycosides. Numerous cardiac glycosides including digitoxin, digoxin, bufalin, ouabain, oleandrin have been explored for their antitumor properties, and have demonstrated remarkable antitumor effects in a variety of cancer types (183–185). The effective use of natural compounds for breast cancer management is currently hindered by several challenges, such as toxicity, low bioavailability, absorption, and targeted delivery issues (186).

In this context, Winnicka et al. created a nanoscaffold of two cardiac glycosides, digoxin and proscillaridin A and G3 PAMAM-NH2 dendrimers. The antiproliferative effects, cytotoxicity, and apoptosis-inducing ability of the glycoside dendrimer nanocomplex were assessed in breast cancer cells. The findings indicate that the nanocomplex G3 dendrimer augments the cytotoxic effects of digoxin and proscillaridin A in MCF-7 and MDA-MB-231 breast cancer cells. Furthermore, nanocomplex-induced apoptosis was markedly more pronounced than that triggered by the pure glycosides digoxin and proscillaridin A (123).

## 7 Limitations and challenges with clinical translation

Even though there has been significant development in nanomedicine research, several challenges make it difficult to use PAMAM dendrimers to deliver natural compounds based anticancer drugs in the clinical trials. Dealing with regulatory hurdles and evaluating safety are among the main challenges. The intricate physiochemical properties of PAMAM dendrimers, including generation number, size variation, surface charge, and functionalization, can profoundly affect their pharmacokinetics and biological interactions. PAMAM dendrimers based nanocarriers require rigorous characterization, long term toxicity analysis, and reliable manufacturing procedures, which complicates regulatory approval (187). A significant restriction pertains to large scale synthesis and reproducibility. PAMAM dendrimers can be synthesized using established divergent or convergent methods, however, the multistep synthesis necessary for higher generations frequently results in elevated production cost and difficulties in preserving structural standardization and uniformity between batches in large scale production (188). These challenges may impact the scalability and industrial viability of PAMAM dendrimers-based formulations.

Potential toxicity with cationic dendrimers and immunological reactions can constitute substantial obstacles. In fact, some early synthesized cationic dendrimers appeared to be significantly toxic to cells in animal models and may induce inflammatory and immunological reactions (189). This is mainly because cationic dendrimers have a high positive charge density that interacts with negatively charged biological membranes and blood cells that leads to hemolysis, cytokine modulation, and complement activation (190–192). Studies have demonstrated that the cytotoxicity of cationic PAMAM dendrimers with modified surface containing amine groups and pro-inflammatory responses of PAMAM dendrimers increases progressively with each generation (192–194). Consequently, altering the surface of PAMAM dendrimers can modify the cytotoxicity and immunogenic properties of the nanomolecules and enhance their biocompatibility (195). The capacity to alter core, branches, and surface of PAMAM dendrimers renders them adaptable for clinical use and plausible therapeutic option for cancer (196).

Moreover, the long term biodistribution and elimination of PAMAM dendrimers *in vivo* necessitate additional research. Experimental investigations indicate that lower generation dendrimers are predominantly eliminated through renal excretion, while higher generation dendrimers may accumulate in organs including liver and spleen due to absorption by the reticuloendothelial system (197, 198). Despite the observed slow elimination in animal models, extensive long term pharmacokinetic and toxicological data remain limited. Thus, despite promising preclinical results suggesting enhanced solubility, stability, and targeted delivery of natural compounds in breast cancer models, PAMAM dendrimers based nanocarriers have not achieved widespread clinic use. Overcoming regulatory, manufacturing, and safety challenges will be crucial to advancing these nanocarriers towards clinical application in breast cancer treatment.

## 8 Conclusion and future perspective

PAMAM dendrimer possess considerable potential for many biomedical application, because of their modifiable physiochemical characteristics, which enhance interactions with cell membranes and permit cellular entry. The nanoscale dimensions, customizable surface properties, interactions with cell membranes and pharmaceuticals, internal cavities, and various other attributes render PAMAM dendrimers highly suitable carriers for drug delivery systems. The functionalization of these nanocarriers through the incorporation of additive molecules improves the solubility, stability and bioavailability of diverse bioactive compounds. Owing to the limited and suboptimal cellular transfection efficiency of natural compounds, specialized formulations have been developed to impede tumor progression and metastasis in patients with breast cancer. In addition to increasing stability and solubility, PAMAM nanocarriers have the potential to extend their half-life and achieve site-specific delivery in breast cancer cells.

However, concerns regarding PAMAM dendrimer mediated natural compounds have not been satisfactorily addressed in clinical translation. A significant limitation of these nanocarriers is their minimal capability to encapsulate bioactive compounds. Custom-designed nanocarriers coupled with certain ligands may allow loaded phytoconstituents to operate at minimal dosages. PAMAM dendrimers can induce significant anticancer effects upon cellular entrance, however as mentioned previously, having cytotoxicological issues. The challenges associated with bioavailability of natural compounds and toxicity of PAMAM dendrimers must be addressed to enhance the anticancer efficacy of natural compounds.

Future research should be focused on clarify the anticancer properties of PAMAM dendrimers, specifically focusing on enhancing their internalization mechanisms and evaluating their cytotoxic efficacy. Identifying these pathways may offer new approaches to minimize the associated cytotoxicity by modulating the cellular entry mechanisms. This innovative method provided substantial benefits beyond the existing emphasis on altering dendrimer surface characteristics, thereby enhancing their therapeutic use in drug delivery approaches and breast cancer treatment.
